# Mendelian Randomization With Refined Instrumental Variables From Genetic Score Improves Accuracy and Reduces Bias

**DOI:** 10.3389/fgene.2021.618829

**Published:** 2021-03-17

**Authors:** Lijuan Lin, Ruyang Zhang, Hui Huang, Ying Zhu, Yi Li, Xuesi Dong, Sipeng Shen, Liangmin Wei, Xin Chen, David C. Christiani, Yongyue Wei, Feng Chen

**Affiliations:** ^1^Department of Biostatistics, Center for Global Health, School of Public Health, Nanjing Medical University, Nanjing, China; ^2^China International Cooperation Center for Environment and Human Health, School of Public Health, Nanjing Medical University, Nanjing, China; ^3^Jiangsu Key Lab of Cancer Biomarkers, Prevention and Treatment, Collaborative Innovation Center for Cancer Personalized Medicine, Nanjing Medical University, Nanjing, China; ^4^State Key Laboratory of Reproductive Medicine, Nanjing Medical University, Nanjing, China; ^5^Department of Biostatistics, University of Michigan, Ann Arbor, MI, United States; ^6^Department of Epidemiology and Biostatistics, School of Public Health, Southeast University, Nanjing, China; ^7^Department of Environmental Health, Harvard T.H. Chan School of Public Health, Boston, MA, United States; ^8^Division of Pulmonary and Critical Care Medicine, Department of Medicine, Massachusetts General Hospital, Harvard Medical School, Boston, MA, United States

**Keywords:** Mendelian randomization, multiple correlated instrumental variables, genetic score, metabolomics, educational attainment

## Abstract

Mendelian randomization (MR) can estimate the causal effect for a risk factor on a complex disease using genetic variants as instrument variables (IVs). A variety of generalized MR methods have been proposed to integrate results arising from multiple IVs in order to increase power. One of the methods constructs the genetic score (GS) by a linear combination of the multiple IVs using the multiple regression model, which was applied in medical researches broadly. However, GS-based MR requires individual-level data, which greatly limit its application in clinical research. We propose an alternative method called Mendelian Randomization with Refined Instrumental Variable from Genetic Score (MR-RIVER) to construct a genetic IV by integrating multiple genetic variants based on summarized results, rather than individual data. Compared with inverse-variance weighted (IVW) and generalized summary-data-based Mendelian randomization (GSMR), MR-RIVER maintained the type I error, while possessing more statistical power than the competing methods. MR-RIVER also presented smaller biases and mean squared errors, compared to the IVW and GSMR. We further applied the proposed method to estimate the effects of blood metabolites on educational attainment, by integrating results from several publicly available resources. MR-RIVER provided robust results under different LD prune criteria and identified three metabolites associated with years of schooling and additional 15 metabolites with indirect mediation effects through butyrylcarnitine. MR-RIVER, which extends score-based MR to summarized results in lieu of individual data and incorporates multiple correlated IVs, provided a more accurate and powerful means for the discovery of novel risk factors.

## Introduction

Observational studies have long been utilized to detect associations between the exposures of interest and the risk of complex diseases. However, the estimated effects are typically biased and causality cannot be directly inferred because of unobserved confounders or reverse causality (Ebrahim and Davey Smith, 2008). Double-blind randomized controlled trials with perfect adherence, which use randomization allocation to avoid potential confounding, are often considered as the gold standard to infer causality (Bothwell et al., 2016). However, logistical difficulties limit the use in real-world studies.

**FIGURE 1:**
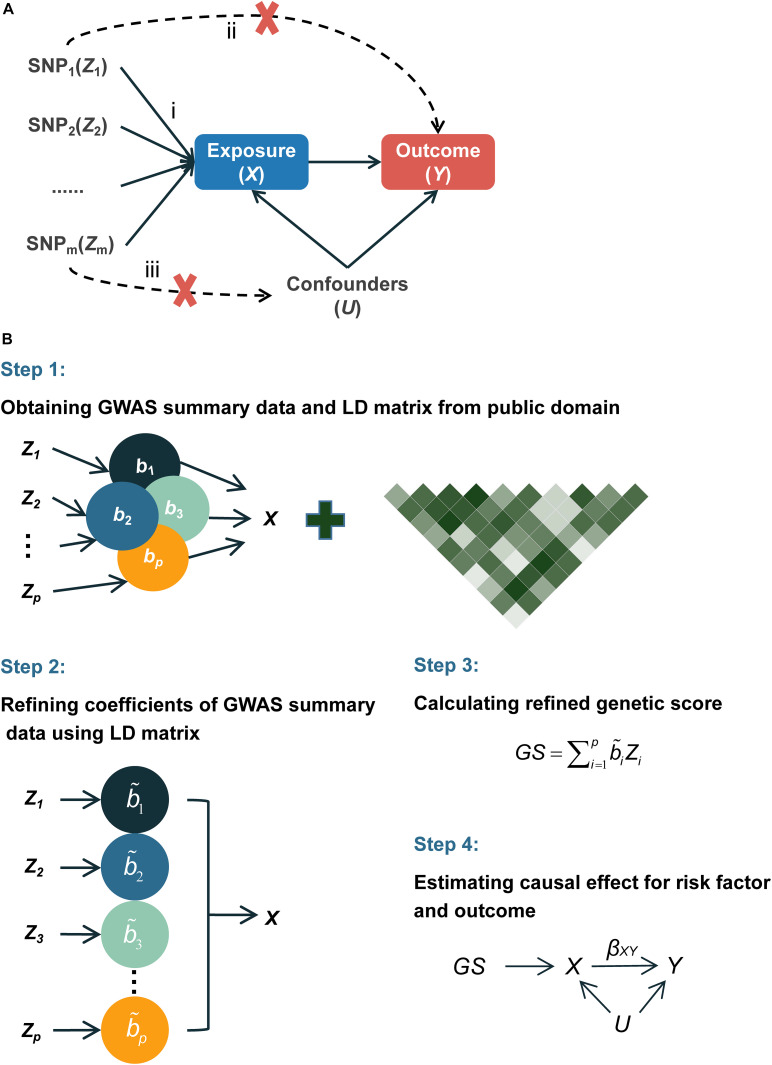
Diagram of Mendelian randomization and flowchart of the proposed MR-RIVER method. **(A)** Mendelian randomization inferring the causal association of the exposure and outcome: (i) IVs are associated with the exposure X; (ii) IVs and outcome *Y* are independent, conditional on exposure *X* and unmeasured confounders *U*; (iii) IVs and confounders *U* are independent. **(B)** Flowchart of the proposed MR-RIVER method for multiple genetic variants in causal inference.

Instrumental variable (IV) analysis provides unbiased causal estimates in the presence of observed and unobserved confounders under certain assumptions (Burgess et al., 2017). A valid IV should (1) be associated with the exposure of interest; (2) not be associated with any confounders of the exposure–outcome association; and (3) affect the outcome only through its impact on the exposure of interest (Figure 1A; Martens et al., 2006). Because human germline genetic variants usually form at fertilization and remain unchanged after birth (Ference et al., 2019), they are less likely to be correlated with the environmental or clinical factors but can be correlated with susceptibility to these factors that are associated with outcomes and thus are ideal candidates for IVs.

Mendelian randomization (MR), which uses genetic variants as IVs, has emerged recently as a powerful tool to estimate the causal effects of risk factors in observational settings (Smith and Ebrahim, 2003; Yavorska and Burgess, 2017; Burgess and Labrecque, 2018; Bowden et al., 2019) and has been increasingly used in genome-wide association studies (GWAS) (Welter et al., 2014; Burgess et al., 2015; Pickrell et al., 2016). However, as a single variant typically explains only a small proportion of variability, a large sample size is often required to power the traditional MR (Pierce et al., 2011). A variety of generalized MR methods have been proposed to integrate results arising from multiple IVs in order to increase power (Burgess and Thompson, 2013; Burgess et al., 2013). These methods include generalized summary-data-based Mendelian randomization (GSMR) (Zhu et al., 2018) and inverse-variance weighted method (IVW) (Burgess et al., 2013, 2016). GSMR integrates estimates from single IVs by using a generalized least-square approach (Zhu et al., 2018), whereas IVW combines estimates by using weights based on the variance–covariance matrix (Burgess et al., 2016). However, these existing methods are based on the summarized results of single-variant analysis and commonly prune IVs based on linkage disequilibrium to obtain relatively independent IVs, resulting in loss of information. Even with adjustment of the correlation structure, the results may still be inefficient. Notably, Burgess et al. (2017) introduced a multivariate regression method, which regresses the exposure factor on multiple IVs at the first stage to construct genetic scores (GSs). GS can be viewed as a linear combination of multiple IVs weighted by the strength of the association between an IV and the exposure, adjusted for all the other IVs. In the ensuing MR analysis, GS will be passed along as a single IV. The method was recently implemented in a study of *ACLY* and cardiovascular disease which incorporated multiple germline genetic variants (IVs) to construct GS as single IV and further inferred the causal relationship between *ACLY* inhibitors and the reduced risk of cardiovascular disease (Ference et al., 2019).

Thus, we propose an alternative method called Mendelian Randomization with Refined Instrumental Variable from Genetic Score (MR-RIVER) (Figure 1B) to construct a genetic score summarizing multiple genetic variants based on summarized results rather than individual-level data. Our method, which accounts for correlations among multiple genetic variants by borrowing linkage disequilibrium (LD) information from public databases (such as 1000 Genomes Project), provides a useful framework to integrate estimates obtained by using various genetic IVs and improves the performance of the summarized genetic score for the correlated genetic variants. Simulation studies suggested improved performance of our proposed method, compared to GSMR and IVW. We further applied the proposed method to estimate the effects of blood metabolites on educational attainment, by integrating results from several publicly available resources (Shin et al., 2014; Okbay et al., 2016).

## Method

### MR-RIVER Algorithm

We propose a method to infer the causal relationship between risk factor *X* (e.g., blood metabolites) and outcome *Y* (e.g., years of schooling) given a set of IVs, denoted by **Z** = (*Z*_1_, *Z*_2_, …, *Z*_*p*_) (e.g., a set of genetic variants). The major components of our framework are depicted in Figure 1A. More specifically, we use *b*_*XZ_i*_, along with standard error *se*(*b*_*XZ*_*i*__), to quantify the association of each *Z*_*i*_ with the risk factor *X* from the traditional single-locus association analysis model, and likewise for *b*_*YZ_i*_ and *se*(*b*_*YZ*_*i*__) for each *Z*_*i*_ with the outcome *Y*.

The unified weighted GS incorporating multiple IVs could be estimated by the linear combination of multiple IVs:

(1) $$G\,S=\sum_{i=1}^{p}{\tilde{b}}_{X\,Z_{i}}\,Z_{i}$$

Where ${\tilde{b}}_{X\,Z_{i}}$ denotes the direct effect of *Z*_*i*_ on *X* after controlling for the other IVs that derived from multivariable regression. However, in practice, the published-available summarized data were derived from single-variant analysis; it is unlikely to get genetic association estimates from a multivariable regression model in a large independent dataset due to issues of practicality and confidentiality of data sharing on such a large scale. Here, we propose an estimator by borrowing the idea of coefficient decomposition to estimate ${\tilde{b}}_{X\,Z_{i}}$ by using summarized results rather than individual-level data.

Specifically, under the assumption that (*X*, **Z**) follow a multivariate normal distribution, regressing *X* on each *Z*_*i*_ will yield an estimate of *b*_*XZ_i*_. Without loss of generality, we assume that there is a linear relationship between *X* and **Z**. As *E*(*X*|*Z*_*i*_) = *b*_0_ + *b*_*XZ*_*i*__*Z*_*i*_, *b*_*XZ_i*_ represents the total effect of *Z*_*i*_ on *X*. After adjusting the effect of all the other IVs, the relationship between *X* and *Z*_*i*_ can be expressed as $\begin{array}{c} E\,\left(X|Z_{1},\cdots ,Z_{p}\right)=b_{0}+{\tilde{b}}_{X\,Z_{1}}\,Z_{1}+\cdots +{\tilde{b}}_{X\,Z_{p}}\,Z_{p} \end{array}$, where ${\tilde{b}}_{X\,Z_{i}}$ is the direct effect of *Z*_*i*_ on *X* under the control of other IVs. Therefore, *b*_*XZ*_*i*__ can be decomposed into the direct effect and indirect effect via other correlated IVs:

(2) $$b_{X\,Z_{i}}={\tilde{b}}_{X\,Z_{i}}+\sum_{j\neq i}^{p}{\tilde{b}}_{X\,Z_{j}}\,\theta_{Z_{j}\,Z_{i}}$$

Here, θ_*Z_j Z_i*_ is the regression coefficient of *Z*_*j*_ on *Z*_*i*_, and ${\tilde{b}}_{X\,Z_{i}}$ is the direct effect of *Z*_*i*_ on *X*, after controlling for the other IVs. Equation 2 can be rewritten as:

(3) $${\tilde{\text{b}}}_{X\,Z}=\theta^{-1}\,{\text{b}}_{X\,Z}$$

where **b**_*XZ*_ is the *p*-length vector containing *b*_*XZ*_*i*__, ${\tilde{\text{b}}}_{X\,Z}$ is the vector of refined coefficients ${\tilde{b}}_{X\,Z_{i}}$, and **θ** is a *p* × *p* matrix with θ_*Z_j Z_i*_ being the *ij*-th entry. It follows that $\theta_{Z_{i}\,Z_{j}}=\rho_{Z_{j}\,Z_{i}}\,\sqrt{v\,a\,r\,\left(Z_{j}\right)\,/\,v\,a\,r\,\left(Z_{i}\right)}$ where *ρ_Z_j Z_i_* is the correlation between *Z*_*j*_ and *Z*_*i*_, *var*(*Z_i_*) is the variance of *Z_*i*_. var*(*Z_i_*) and *ρ_Z_jZ_i_* can be obtained from the public GWAS resources (e.g., 1000 Genomes Project).

We note that Eq. 3 is crucial as it enables us to compute GS defined in Eq. 1 with only summary data, in lieu of individual-level data. With GS as a single IV, we can estimate the association between the risk factor *X* and outcome *Y* with:

(4) $${\hat{\beta }}_{X\,Y}=\frac{\beta_{Y\,G\,S}}{\beta_{X\,G\,S}}=\frac{c\,o\,v\,\left(Y,G\,S\right)}{c\,o\,v\,\left(X,G\,S\right)}=\frac{c\,o\,v\,\left(Y,\sum_{i}^{p}{\tilde{b}}_{X\,Z_{i}}\,Z_{i}\right)}{c\,o\,v\,\left(X,\sum_{i}^{p}{\tilde{b}}_{X\,Z_{i}}\,Z_{i}\right)}=\frac{\sum_{i}^{p}{\tilde{b}}_{X\,Z_{i}}\,c\,o\,v\,\left(Y,Z_{i}\right)}{\sum_{i}^{p}{\tilde{b}}_{X\,Z_{i}}\,c\,o\,v\,\left(X,Z_{i}\right)}=\frac{\sum_{i}^{p}{\tilde{b}}_{X\,Z_{i}}\,b_{Y\,Z_{i}}\,v\,a\,r\,\left(Z_{i}\right)}{\sum_{i}^{p}{\tilde{b}}_{X\,Z_{i}}\,b_{X\,Z_{i}}\,v\,a\,r\,\left(Z_{i}\right)}$$

As mentioned by Burgess et al. (2016), *var*(*Z*_*i*_) is approximately proportional to 1/*var*(*b*_*YZ*_*i*__); thus, Eq. 4 can be simplified as:

(5) $${\hat{\beta }}_{X\,Y}=\frac{\sum_{i}^{p}{\tilde{b}}_{X\,Z_{i}}\,b_{Y\,Z_{i}}\,/\,v\,a\,r\,\left(b_{Y\,Z_{i}}\right)}{\sum_{i}^{p}{\tilde{b}}_{X\,Z_{i}}\,b_{X\,Z_{i}}\,/\,v\,a\,r\,\left(b_{Y\,Z_{i}}\right)}$$

The asymptotic standard error for ${\hat{\beta }}_{X\,Y}$ can be estimated by the delta method (Thomas et al., 2007):

(6) $$s\,e\,\left({\hat{\beta }}_{X\,Y}\right)=\sqrt{\frac{\sum_{i}^{p}\sum_{j}^{p}\rho_{Z_{i}\,Z\,j}\,{\tilde{b}}_{X\,Z_{i}}\,{\tilde{b}}_{X\,Z_{j}}\,/\,\left(s\,e\,\left(b_{Y\,Z_{i}}\right)\,s\,e\,\left(b_{Y\,Z_{j}}\right)\right)}{{\left(\sum_{i}^{p}{\tilde{b}}_{X\,Z_{i}\,X\,Z_{j}}\,/\,v\,a\,r\,\left(b_{Y\,Z_{i}}\right)\right)}^{2}}}$$

The association between *X* and *Y* can be further tested by using the Wald test statistic $u={\hat{\beta }}_{X\,Y}\,/\,s\,e\,\left({\hat{\beta }}_{X\,Y}\right)$, which asymptotically follows a standard normal distribution under the null hypothesis.

We stress that, though Eqs.5, 6 resemble the estimator proposed in Burgess et al. (2017), our estimator differs from that in Burgess et al.’s (2017) required individual data, while our estimator, with the introduction of the refined estimates in Eq. 3, can be computed even with the summary data. Therefore, our estimator is applicable in more broad settings, where only summary data are available. Simulations have confirmed the utility of our method.

### Design of Statistical Simulations

Two sets of simulation studies were designed to investigate MR-RIVER.

#### Evaluation of the Estimates of the Refined Coefficients of IVs on *X*

We generated six IVs, *Z*_1_, *Z*_2_, …, *Z*_6_, from a multivariate normal distribution *MVN* (**0**, **Σ**), where **Σ** is a correlation matrix with an equal correlation structure. We varied the correlation coefficient and set it to be 0, 0.1, 0.3, 0.5, 0.7, and 0.9, corresponding to various scenarios: from the independent case to the highly correlated case. We generated *X* using the following models:

(7) $$\begin{array}{c} X_{i}=\Sigma_{j=1}^{6}Z_{i\,j}\,{\tilde{b}}_{j}+\varepsilon_{X\,i}\\ {\tilde{b}}_{j}∼N\,\left(\mu ,1\right),\mu =-1,-0.5,0.5,1,1.5,2\\ \varepsilon_{X_{i}}∼N\,\left(0,1\right) \end{array}$$

The sample size was fixed at 1,000. In addition, we simulated 5,000 additional individuals to provide an external correlation structure for IVs. For each simulation configuration, 2,000 datasets were produced.

We first regressed *X* on each *Z*_*j*_ separately to obtain the summarized effect of *Z*_*j*_ on *X*, and based on these results, we applied Eq. 2 to obtain the estimates of the refined coefficients. The estimated refined coefficients, along with the corresponding standard errors, were compared to those from traditional GWAS summarized results under different correlation structures and effect sizes of *Z*.

#### Investigation of the Statistical Properties of MR-RIVER

Let *X*_*i*_ and *Y*_*i*_ denote the exposure and outcome variables of the *i*th subject, and *Z*_*ij*_ the *j*th IV (*j* = 1, …, *J*). The data were generated from the following model:

(8) $$\begin{array}{c} Z_{i}∼M\,V\,N\,\left(0,\text{Σ}\right),b_{j}∼U\,\left(0,0.5\right)\\ X_{i}=\Sigma_{j=1}^{J}Z_{i\,j}\,b_{j}+\varepsilon_{X\,i}\\ Y_{i}=X_{i}\,b_{X\,Y}+\varepsilon_{Y\,i}\\ w\,h\,e\,r\,e\;\varepsilon_{X\,i}∼N\,\left(0,v\,a\,r\,\left(\Sigma_{j=1}^{J}Z_{i\,j}\,b_{j}\right)\,\left(R_{Z\,X}^{-2}-1\right)\right)\\ a\,n\,d\;\varepsilon_{Y\,i}∼N\,\left(0,v\,a\,r\,\left(X_{i}\,b_{X\,Y}\right)\,\left(R_{X\,Y}^{-2}-1\right)\right) \end{array}$$

where **Σ** is the correlation matrix of IVs with an equal correlation structure. We varied the correlation parameter from 0 to 0.9 by 0.1. Each IV explains 0.005 of the variance of *X*, and we considered *J* = 5, 10, 15, 20. Moreover, $R_{Z\,X}^{2}$ is the proportion of variance of *X* explained by all IVs, which was set to be 0.025, 0.05, 0.075, and 0.1, while $R_{X\,Y}^{2}$ is the proportion of variance of *Y* explained by *X*, which was set to be 0.05, 0.1, 0.15, and 0.2. Sample sizes for the IV-exposure association study (*N*_1_) and the IV-outcome association study (*N*_2_) were set to be 1,000 and 1,500, respectively. In addition, 5,000 (*N*_3_) individuals were generated to provide an external correlation structure for genetic variants.

For each parameter configuration, a total of 2,000 datasets were produced. Under all the scenarios examined, MR-RIVER was found to outperform GSMR and IVW by maintaining the Type I error, possessing more statistical power, as well as having smaller biases and mean squared errors.

## Results

### Statistical Properties of Refined Coefficients

We investigated the accuracy of refined coefficients. With the obtained correlation structure of IVs from the internal analysis set, the estimated refined coefficients (along with the standard errors) based on the summarized results were in consistent with the corresponding estimates from multivariable regression (Supplementary Figures 1A,B), suggesting that the estimates of the refined coefficients were unbiased.

**FIGURE 2:**
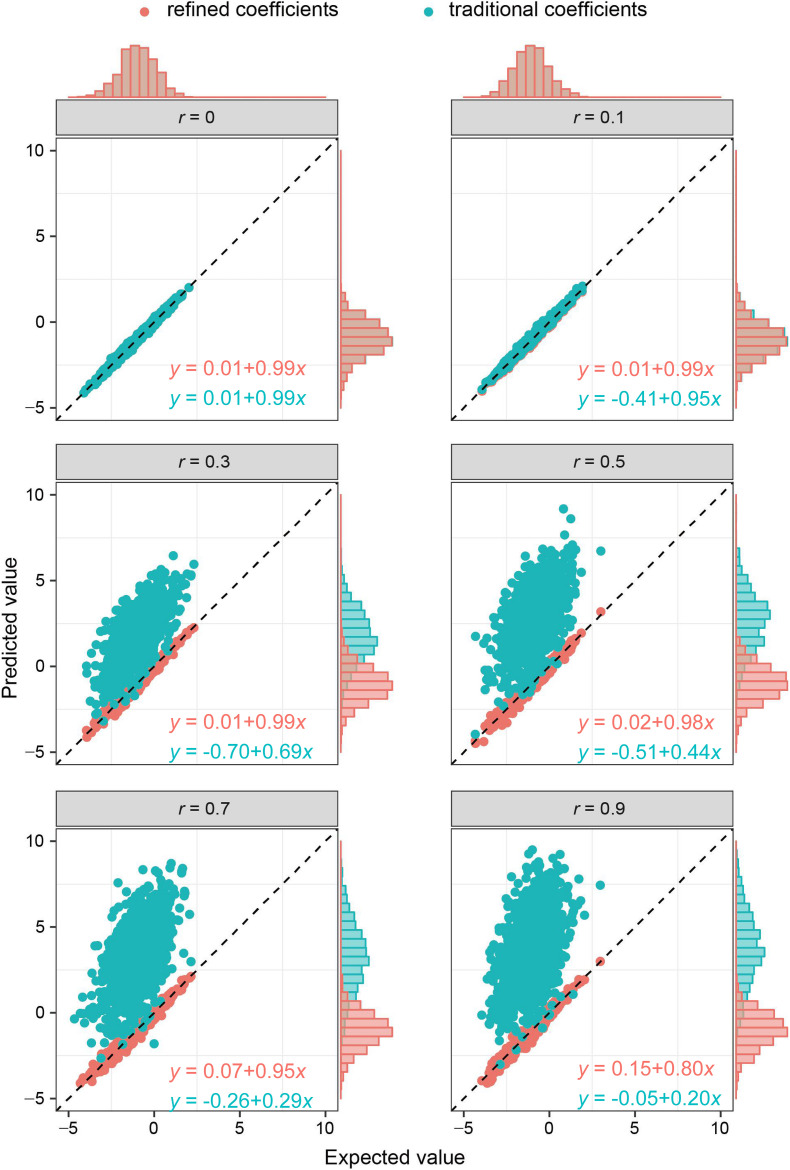
Comparison of refined and traditional coefficients under different correlation structures. Expected values are the regression coefficients obtained from the multivariable regression model with all the variables used to generate dependent variable Y plotted against predicted values obtained from the refined method (refined coefficients) and traditional single-locus analyses (traditional coefficients). Refined and traditional coefficients were compared with the bias from expected coefficients under different correlation structures through a regression model. Red equation represents the relationship between expected coefficients and refined coefficients, and green equation represents traditional coefficients.

**FIGURE 3:**
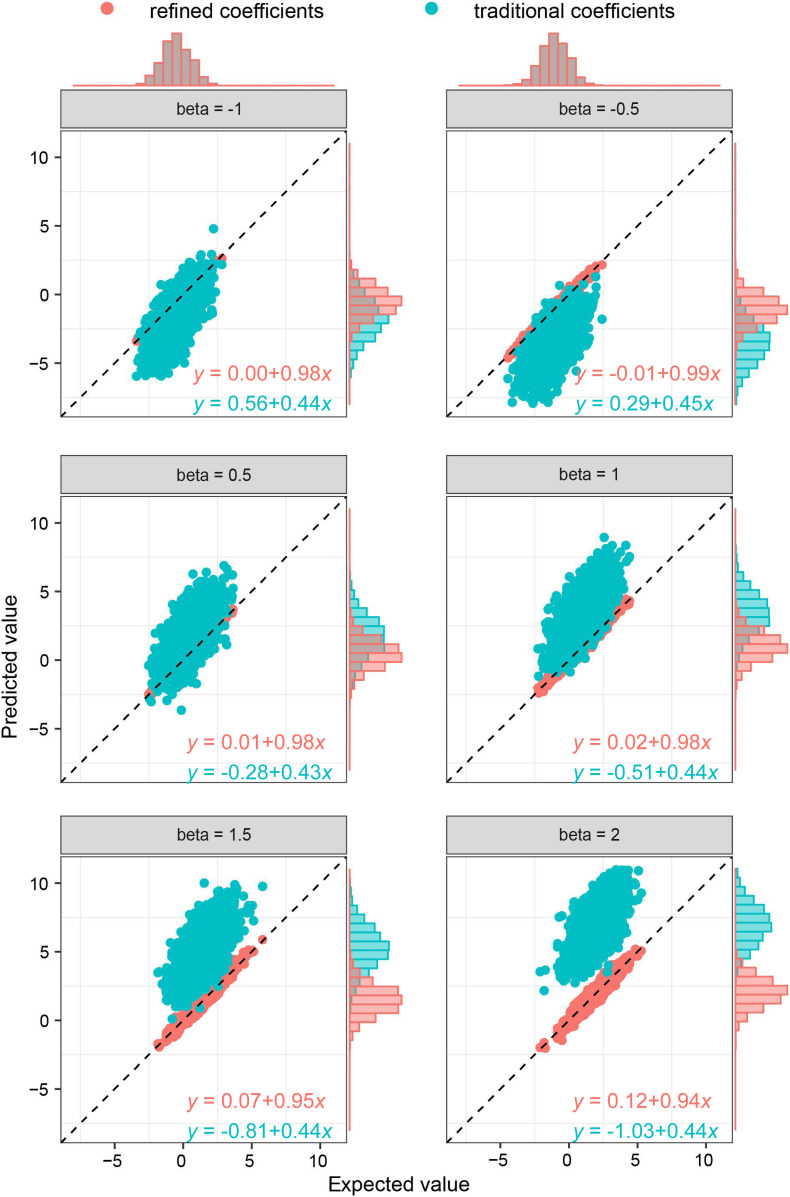
Comparison of refined and traditional coefficients under different effect sizes. Expected values are the regression coefficients obtained from the multivariable regression model with all the variables used to generate dependent variable *Y* plotted against predicted values obtained from the refined method (refined coefficients) and traditional single-locus analyses (traditional coefficients). Refined and traditional coefficients were compared with the bias from expected coefficients under different effect sizes through a regression model. Red equation represents the relationship between expected coefficients and refined coefficients, and green equation represents traditional coefficients.

As the key of the approach lies in borrowing the correlation information from public resources, we further evaluated the method by obtaining the correlation structure from the simulated external samples. According to different levels of correlation among IVs, refined coefficients outperformed traditional coefficients obtained from single-locus analysis, especially when the correlations among IVs were relatively high (Figure 2). Similarly, under the specific correlation structure (with a correlation coefficient of 0.5), refined coefficients remained approximately unbiased, while traditional coefficients showed increased biases with increased effect sizes (Figure 3).

### Statistical Properties of MR-RIVER

**FIGURE 4:**
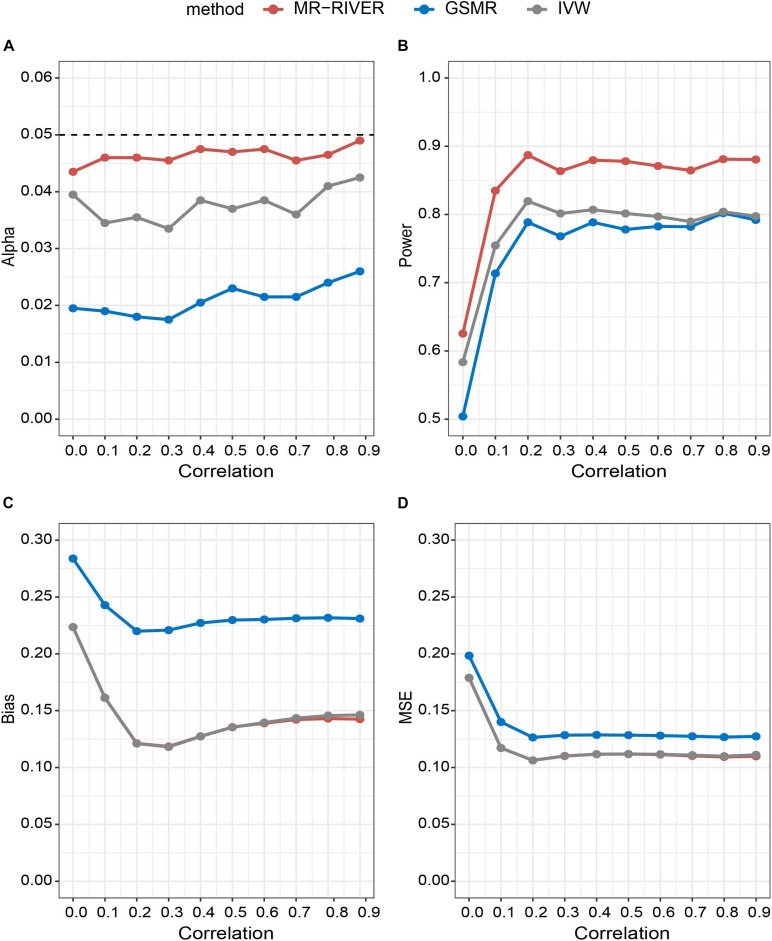
Statistical properties of MR methods under different correlations. Correlation between IVs plotted against: **(A)** type I error under the null hypothesis; **(B)** performance of power under the alternative hypothesis with *b*_*xy*_ = 1; **(C)** bias under the alternative hypothesis; and **(D)** mean square error.

With various strengths of correlations among IVs, MR-RIVER maintained the type I error at the 0.05 level, compared to the IVW (with type I error around 0.04) and GSMR (with the most conservative control of the type I error) (Figure 4A). The results held when we varied the sample size (Supplementary Figure 2A) or the number of IVs (Supplementary Figure 3A). Further, increasing correlation strengths among IVs (Figure 4B), or increasing sample size (Supplementary Figure 2B), or increasing the numbers of IVs (Supplementary Figure 3B) led to increased power for all MR methods. Overall, the power of MR-RIVER was higher than that of GSMR and IVW under different parameter settings.

Estimates of *b*_*xy*_ from the three MR methods were approximately unbiased, while the biases of the MR-RIVER and IVW estimates were lower than that of the GSMR estimate (Figure 4C). The bias increased with the increased effect size (Supplementary Figure 4) and so was true for the MSE (Figure 4D). MR-RIVER and IVW had lower biases and MSEs, compared to GSMR.

## Real Data Application

### Motivation

Educational attainment is moderately heritable and has been recognized as a proxy phenotype for intelligence, cognition, and neuropsychiatric disorders (Berry et al., 2006; Esch et al., 2014). Discovery of the causal factors linking to the educational attainment could shed light on the biological pathways underlying human behavioral and health-related outcomes (Rietveld et al., 2013). Blood metabolites, which closely represent the physiological status of an organism, have garnered significant interest in biomedical research (Simpson et al., 2016). However, few studies have focused on a causal relationship between metabolites and educational attainment in the presence of multiple IV variables. Taking advantage of the proposed MR-RIVER, this application aims to systematically evaluate the causal relationship between blood metabolites and educational attainment using multiple GWAS summary results.

### Materials

Genome-wide association studies summary results for educational attainment were obtained based on various studies from the Social Science Genetic Association Consortium^1^ (Berry et al., 2006; Rietveld et al., 2013). Educational attainment was measured as the year of schooling completed (EduYears) among 293,723 individuals (with a mean of 14.3 years) (Supplementary Table 1). Approximately, 9.3 million SNPs were included in the association analysis, and minor allele frequencies were obtained from the 1000 Genomes Project. Details of the SNPs included in our analysis are displayed in Supplementary Table 2.

Summary results of quantitative trait locus (QTL) analysis of SNPs on corresponding metabolites were obtained from 7,824 European adult individuals (Supplementary Table 3) (Shin et al., 2014). Specifically, the metabolite QTL (mQTL) data contained all of the summarized association statistics for 529 metabolites with *P*-values less than 1 × 10^–5^^2^. A total of 196 metabolites out of 529 (37%) were unknown because their chemical identity was not yet determined at the time of analysis. Detailed information of metabolites can be found in Supplementary Table 4.

### MR Analysis Results

**FIGURE 5:**
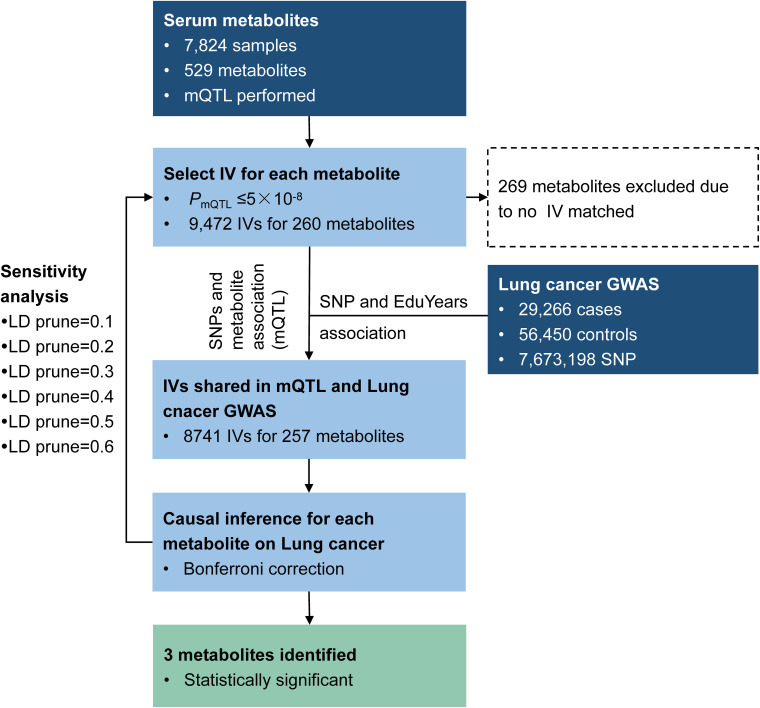
Study workflow for educational attainment MR analysis.

We applied the method to explore the causal effect of blood metabolites on educational attainment as depicted in Figure 5. Based on assumption (1) of IV, SNPs were required to have an mQTL relationship with the corresponding metabolites with *P-values* less than 5 × 10^–8^. As a result, 9,472 SNPs were selected as IVs, matched with 260 metabolites. Among these, 9,329 SNPs were available in the educational attainment GWAS.

**FIGURE 6:**
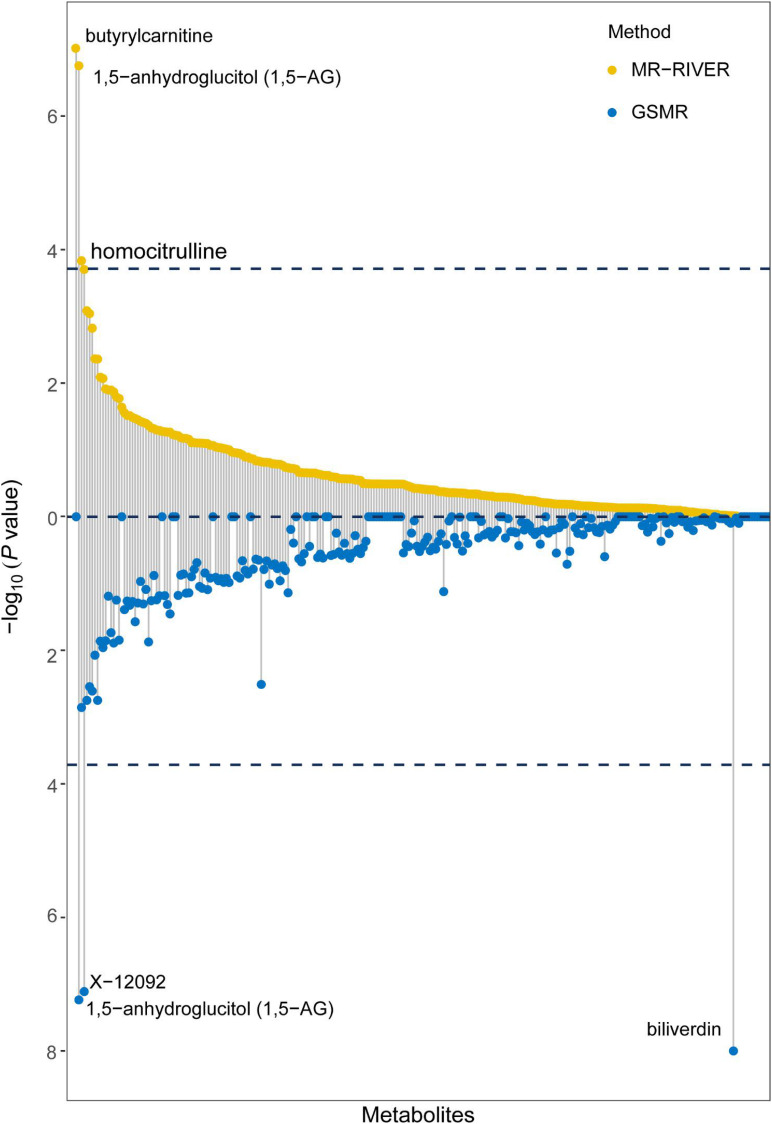
MR-RIVER and GSMR analysis for causal association between metabolites and educational attainment. Relationship between individual metabolites with –log_10_ (*P*-value) of the association. Upper yellow values represent MR-RIVER results, and lower blue values represent GSMR results. Associated metabolites are annotated.

Causal inference for each metabolite on quantitative education years was evaluated through MR-RIVER and GSMR. To obtain sufficient IVs to increase the power of MR, IVs were pruned by LD at 0.5; The HEIDI-outlier test was used to detect pleiotropic SNPs and remove them from the MR analysis; see Figure 6. Bonferroni correction was used to control for false positives. MR-RIVER identified three metabolites associated with education years: butyrylcarnitine (*b_*xy*_* = −0.043, *P* = 1.08 × 10^–7^), 1,5-anhydroglucitol (1,5-AG) (*b_xy_* = −0.192, *P* = 1.77 × 10^–7^), and homocitrulline (*b_xy_* = −0.269, *P* = 1.47 × 10^–4^). GSMR identified biliverdin (*b_*xy*_* = −0.028, *P* = 2.92 × 10^–15^), 1,5-AG (*b_xy_* = −0.183, *P* = 5.83 × 10^–8^), and an unknown metabolite, X-12092 (retention time, 1.130; mass-to-charge ratio, 144.2; spectra, 84.2:0.8) (*b_xy_* = 0.028, *P* = 3.85 × 10^–7^) (Table 1). In addition, sensitivity analyses with different LD prune criteria (0.1–0.7, in 0.1 increments) showed robust results for MR-RIVER, but not for GSMR (Supplementary Tables 5, 6).

**TABLE 1:** Relative bias of imputed datasets with three imputation methods.

Method	Metabolite	*b*_*xy*_	*se of b_*xy*_*	*P-value*
MR-RIVER	Butyrylcarnitine	−0.0430	0.0081	1.08 × 10^–07^
	1,5-Anhydroglucitol (1,5-AG)	−0.1916	0.0367	1.77 × 10^–07^
	Homocitrulline	−0.2687	0.0708	1.47 × 10^–04^
GSMR	Biliverdin	−0.0284	0.0036	2.92 × 10^–15^
	1,5-Anhydroglucitol (1,5-AG)	−0.1838	0.0339	5.83 × 10^–08^
	X-12092	0.0283	0.0056	3.85 × 10^–07^

**b*_*xy*_: causal effect of metabolite and educational attainment.*

**se of b_*xy*_*: standard error of causal effect.*

**P value*: *P*-value of causal effect.*

*X-12092: unknown metabolite (retention time, 1.130; mass-to-charge ratio, 144.2; spectra, 84.2:0.8).*

**FIGURE 7:**
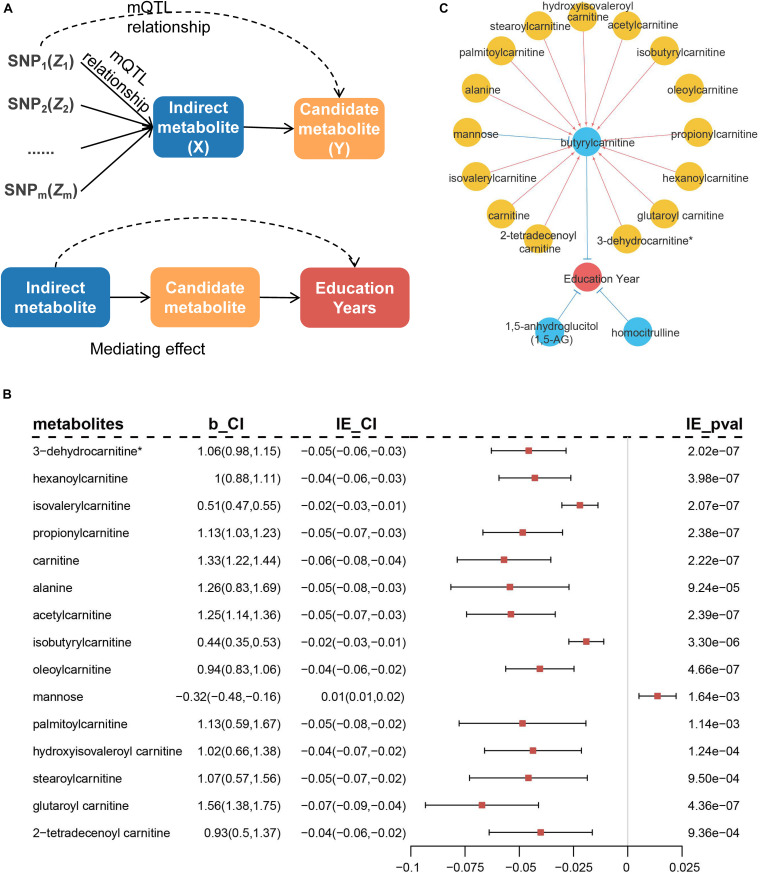
Diagram of MR analysis between metabolites and mediation analysis. **(A)** MR inferring the causal association of remaining metabolites (*X*) on previously identified metabolites (*Y*). Mediation analysis of the rest of metabolites on risk of education years through the identified metabolites. **(B)** Metabolites that indirectly mediate the effect on education years through butyrylcarnitine in mediation analysis. b_CI represents effect of metabolites on butyrylcarnitine and 95% confidence interval (95% CI). IE_CI represents indirect effect of metabolites on education years and 95% CI, and IE_pval represents *P*-values. **(C)** Causal network of blood metabolites on education years. Blue circles indicate metabolites that are directly identified, while yellow circles have indirect effect through blue metabolites. Red lines represent positive effects, and blue lines indicate negative effects.

We performed additional analyses to explore whether the remaining metabolites affected education years through the above-identified candidate metabolites. SNPs associated with the remaining metabolites were treated as IVs to infer potential causal associations between the identified metabolites and remaining metabolites (Figure 7A). The results indicated 28 additional metabolites were associated with the three candidate metabolites. Among these, 24 metabolites (including six unknown metabolites) were associated with butyrylcarnitine, three unknown metabolites were associated with 1,5-AG, and one unknown metabolite was associated with homocitrulline (Supplementary Table 7).

Further, mediation analysis was used to evaluate potential metabolic regulatory pathways for education years by Sobel test (Baron and Kenny, 1986). The 15 metabolites indirectly mediated the effect on education years through butyrylcarnitine (Figure 7B and Supplementary Table 7). Most metabolites were located in the carnitine metabolism pathway (8/15, 53.0%). Blood metabolic biomarkers overall formed a potential causal network (Figure 7C).

## Discussion

We proposed an improved MR approach, MR-RIVER, to combine summarized results of multiple IVs into a single GS and to estimate the unbiased causal effect of a risk factor on an outcome. The publicly accessible summary-level data were obtained from single-locus analyses without consideration of the correlation between IVs. MR-RIVER provides a novel way to refine the effect size of genetic variants account for the correlation based on summary data and makes it efficient to perform summarized data genetic score MR when the correlation between IVs are unignorable. MR-RIVER closely maintains the type I error around the nominal level while it has higher power, lower bias, and smaller variation compared to GSMR and IVW.

Genome-wide association studies uses original GWAS summarized results for IV exposure and IV outcome obtained from single-locus analyses and then derives the causal effect by the generalized least-square approach weighted by the variance–covariance matrix to adjust for correlations among IVs (Zhu et al., 2018). MR-RIVER instead first modifies the summarized results, accounting for correlations among IVs, and then integrates the results. Thus, there are several differences between MR-RIVER and GSMR. First, MR-RIVER adjusts summarized results for each genetic IV by borrowing external LD information to obtain more accurately estimate IV-exposure effect—therefore, MR-RIVER has an advantage in accuracy. Second, MR-RIVER aggregates multiple IVs by weighted linear combination weighted by refined coefficients, which reduces the dimension for IVs and simplifies the following calculation.

Interestingly, MR-RIVER and IVW showed similar performance in bias and MSE. If the weights used to aggregate multiple IVs are equal to the original GWAS summary results (${\tilde{b}}_{X\,Z_{i}}=b_{X\,Z_{i}}$ in Eq. 5), then MR-RIVER is the same as IVW. On the one hand, estimates of MR-RIVER are approximately identical to IVW because point estimates are robust toward the weights (Supplementary Figure 5A). On the other hand, different weights result in different standard errors (Supplementary Figure 5B), which in turn lead to different statistics (Supplementary Figure 5C). This may explain why the bias and MSE of MR-RIVER and IVW are similar, but the performance of power and type I error is different. To summarize, MR-RIVER improves upon IVW and is powerful to infer a causal relationship between an exposure and outcome.

There has been much discussion on the potentials and limitations of MR, as the IV assumptions cannot be fully tested (VanderWeele et al., 2014; Paternoster et al., 2017). Horizontal pleiotropy is a common phenomenon in the human genome that some genetic variants affect the outcome through other traits or pathways rather than exclusively through the risk factor (Solovieff et al., 2013). It is a violation of the instrumental variable assumptions and may induce a major source of potential bias in causal inference. There are several methods are proposed to detect pleiotropy (Slob and Burgess, 2020). The MR-Egger method is able to assess the pleiotropic effects as well as to provide a consistent estimate of the causal effect (Bowden et al., 2017), while the estimates were generally imprecise with low power (Slob and Burgess, 2020). The HEIDI-outlier test was proposed to detect heterogeneity at multiple correlated instruments (Zhu et al., 2018). It will be powerful and valuable when only some proportion of the SNPs have a horizontal pleiotropy effect. In our proposed method, we ensembled the HEIDI-outlier test to detect potential pleiotropy and then remove them from the MR-RIVER analysis.

Notably, after GWAS significant threshold screening, LD prune, and HEIDI-outlier filtering, MR-RIVER analysis suggested three causal metabolites that are associated with education years. The first metabolite is butyrylcarnitine, classified as an acylcarnitine. Previous studies have shown that abnormally increased levels of acylcarnitines, including butyrylcarnitine, are associated with fatty acid oxidation disorders (Jones et al., 2010). Elevated butyrylcarnitine concentration in plasma is associated with short-chain acyl-CoA dehydrogenase deficiency (van Maldegem et al., 2006), which may cause failure to thrive, developmental and cognitive delay, seizures, and neuromuscular (Corydon et al., 2001). Moreover, fatty acid oxidation disorders may lead to mitochondrial dysfunction and further affect the energy supply of the brain (Kölker et al., 2004; Wajner and Amaral, 2015). Therefore, high levels of acylcarnitines may be involved in potential metabolic regulatory pathways affecting cognitive status or brain energy supplement and, in turn, increased education years (mannose→butyrylcarnitine→education years). Mannose easily crosses the blood–brain barrier and is converted to fructose-6-phosphate that enters the glycolytic pathway (Sharma et al., 2014). Cerebral tissue can utilize mannose directly and rapidly from the blood to restore or maintain normal metabolic functions in the absence of glucose (Sloviter and Kamimoto, 1970). Taken altogether, mannose levels appear to be a potential beneficial factor for education years.

The second metabolite, 1,5-AG, is a monosaccharide structurally similar to glucose and is a validated marker of short-term glycemic control (Buse et al., 2003). Low levels of 1,5-AG, indicative of glycemic peak, are associated with dementia and cognitive decline (Rawlings et al., 2017). Finally, elevated homocitrulline, the third metabolite, is structurally similar to but one methylene group longer than citrulline, and impairs bioenergetics in the brain cortex, by reducing velocity of the citric acid cycle and creatine kinase activity. Consequently, it decreases energy production and transfer (Viegas et al., 2009). Therefore, administration of 1,5-AG and homocitrulline may improve educational attainment.

In conclusion, the proposed MR-RIVER method appears to outperform the existing commonly used MR methods. With publicly accessible summary-level data, MR-RIVER provides a more accurate and powerful mean for novel discoveries and identifies several blood metabolites as biomarkers and interventional targets for educational attainment.

## Data Availability Statement

Publicly available datasets were analyzed in this study. This data can be found here: GWAS summary results for education attainment are available at https://www.thessgac.org/data; summary results of quantitative trait locus (QTL) analysis of SNPs on metabolites are available at http://metabolomics.helmholtz-muenchen.de/gwas.

## Author Contributions

LL, RZ, YW, and FC contributed the study design. LL, YW, and RZ performed the statistical analysis and interpretation and drafted the manuscript. YW, LL, RZ, YL, XD, SS, HH, YZ, LW, XC, and DC revised the manuscript. RZ, YW, and FC provided the financial support and study supervision. All authors contributed to the article and approved the submitted version.

## Conflict of Interest

The authors declare that the research was conducted in the absence of any commercial or financial relationships that could be construed as a potential conflict of interest.

## Footnotes

1 https://www.thessgac.org/data

2 http://metabolomics.helmholtz-muenchen.de/gwas
