# Supplementary material for: Mendelian Randomization With Refined Instrumental Variables From Genetic Score Improves Accuracy and Reduces Bias
Source: Front Genet. 2021 Mar 17;12:618829. doi: 10.3389/fgene.2021.618829 (PMC8044958; doi:10.3389/fgene.2021.618829)

**Mendelian randomization with refined instrumental variables from genetic score improves accuracy and reduces bias**

**Supplementary Figure 1. Relationship between refined coefficients and expected values.**

The relationship between predicted values calculated from a modified method based on summary data and expected values calculated from multivariate regression based on individual data for (**a**) coefficients and (**b**) standard error (SE).

**
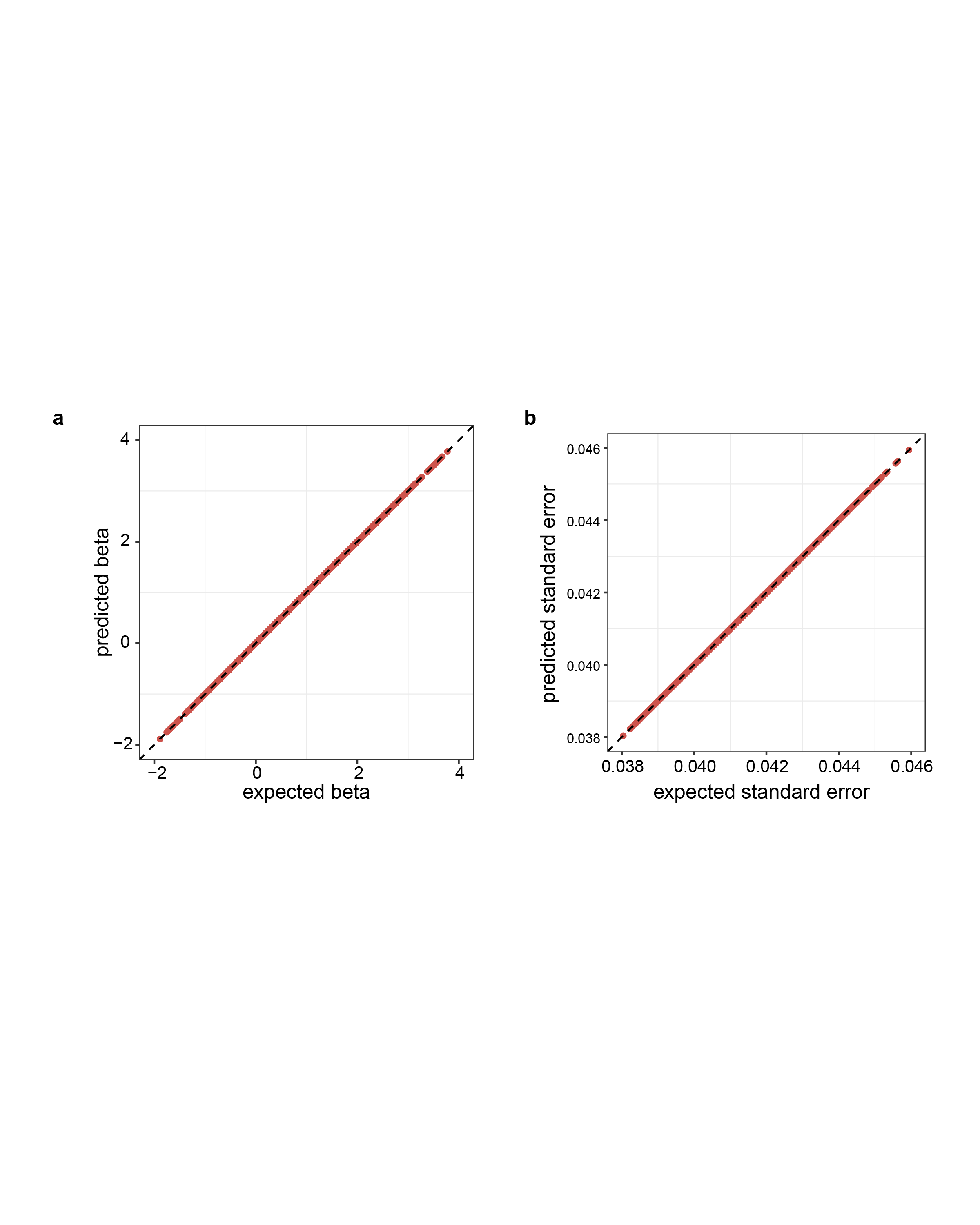
**

**Supplementary Figure 2. Type I error and power under different sample sizes.**

The relationship between sample size and (**a**) corresponding type I error or (**b**) power for MR-RIVER, GSMR, and IVW methods.


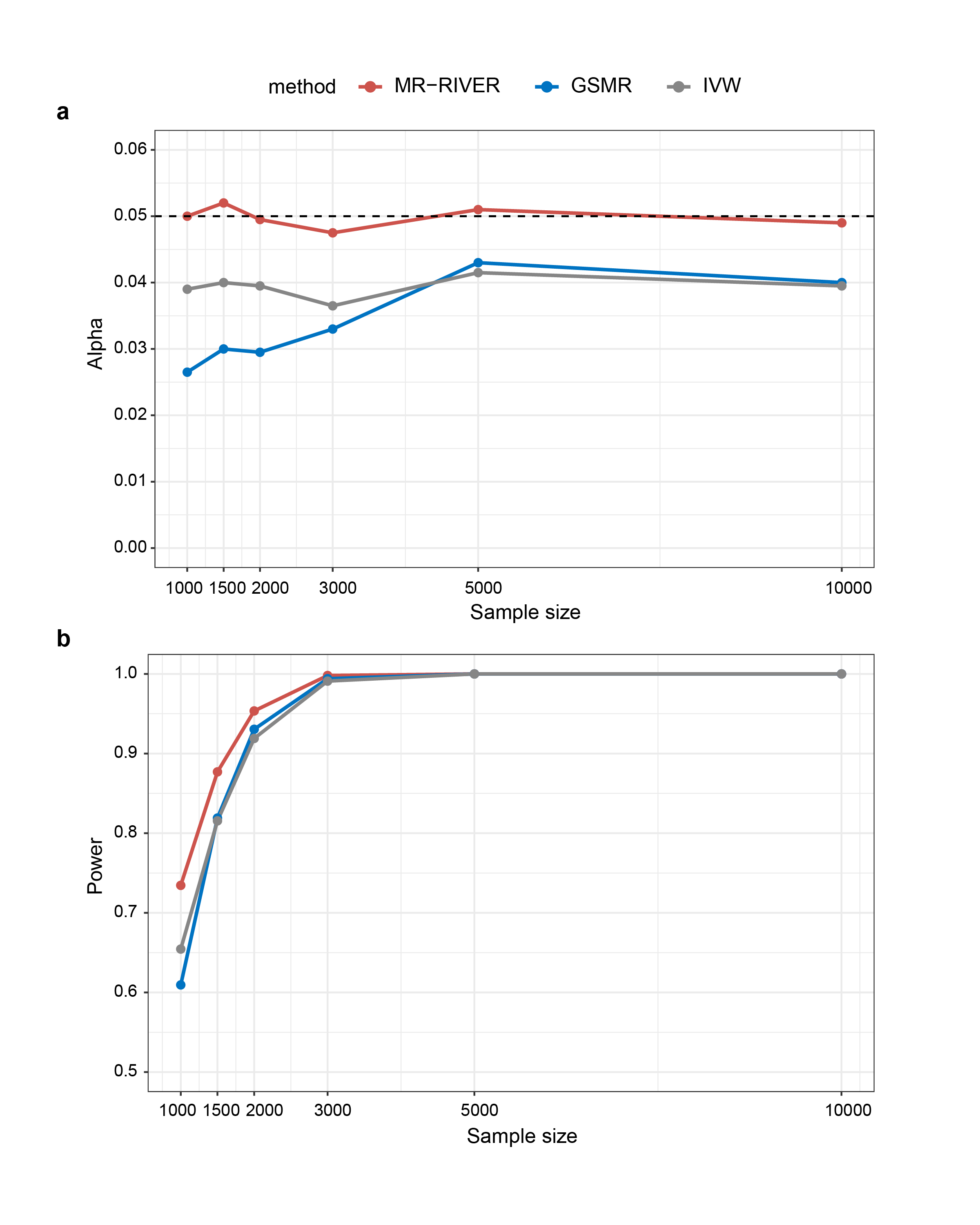


**Supplementary Figure 3. Type I error and power under different numbers of IVs.**

The relationship between number of IVs and (**A**) corresponding type I error or (**B**) power for MR-RIVER, GSMR, and IVW methods.


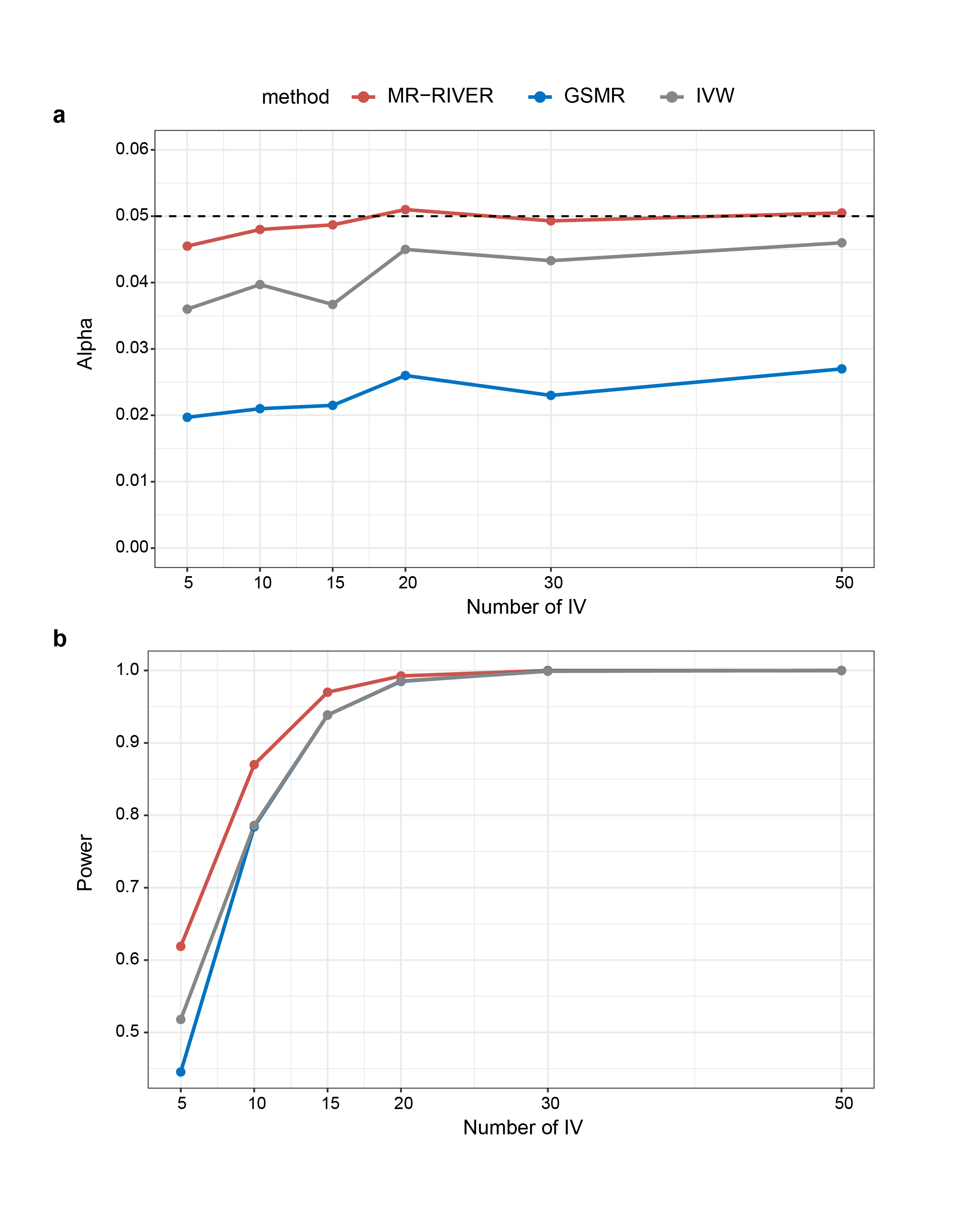


**Supplementary Figure 4. Bias of MR methods under different effect sizes of *b_xy_*.**

Relationship between effect size of *b_xy_* for the simulation and the corresponding bias for MR-RIVER, GSMR, and IVW methods.

**
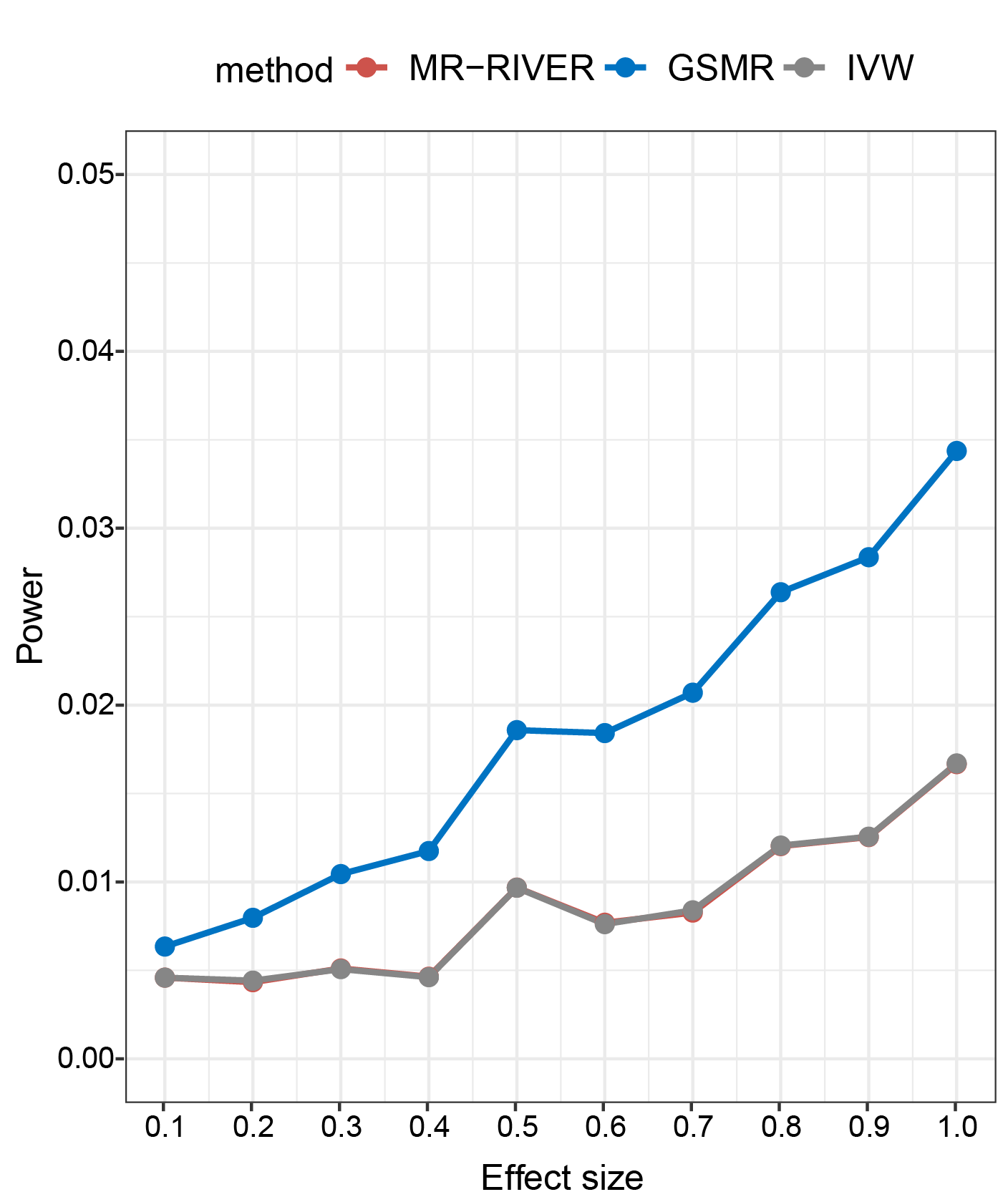
**

**Supplementary Figure 5. Comparison of estimation between MR-RIVER and IVW.**

Relationship between estimates from MR-RIVER and estimates from IVW, including (**A**) causal effect estimates, (**B**) standard error of estimates, and (**C**) Wald test statistic *u*.


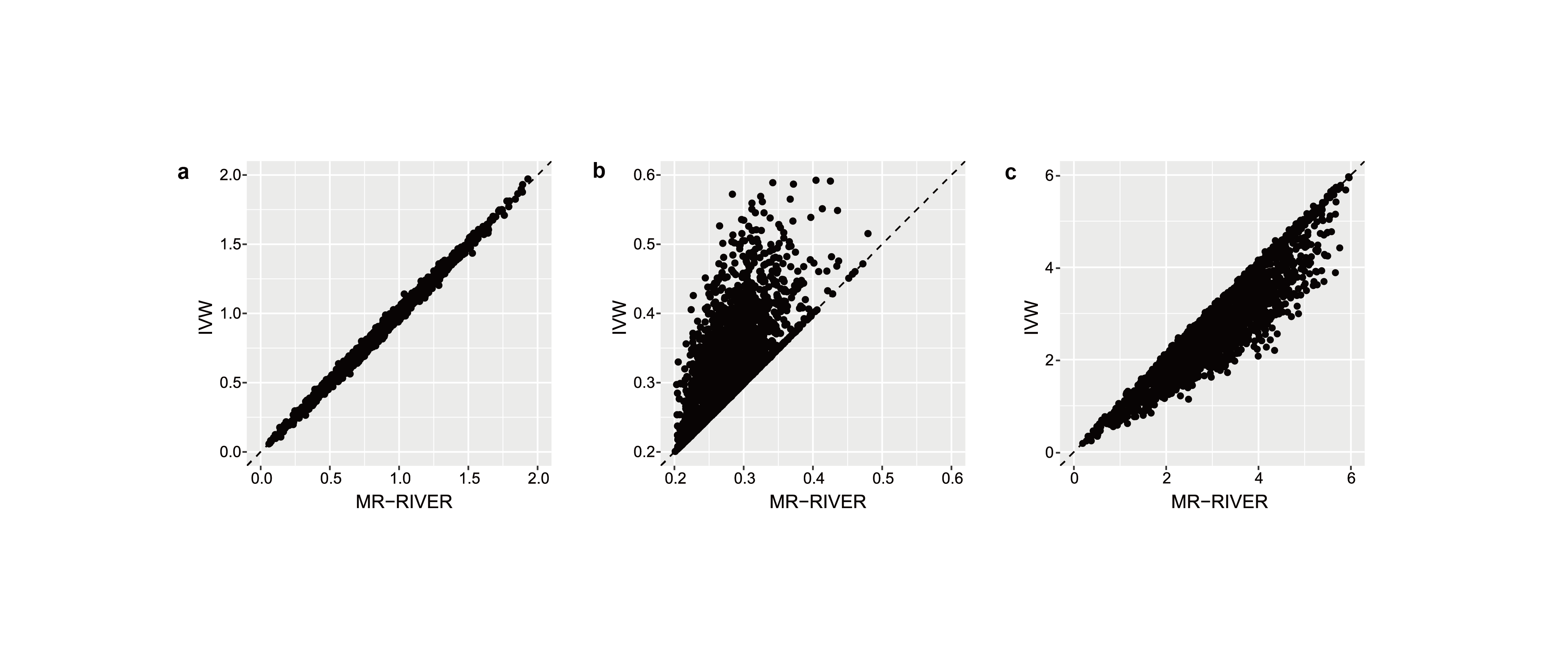

Supplement: Supplementary file 2 [file Data_Sheet_2.doc]
